# Progress in therapeutic targets on podocyte for Alport syndrome

**DOI:** 10.2478/jtim-2024-0005

**Published:** 2024-05-21

**Authors:** Qimin Zheng, Xiangchen Gu, John Cijiang He, Jingyuan Xie

**Affiliations:** Department of Nephrology, Shanghai Ruijin Hospital, Shanghai Jiao Tong University, School of Medicine, Shanghai, China; Institute of Nephrology, Shanghai Jiao Tong University, School of Medicine, Shanghai, China; Department of Medicine, Barbara T. Murphy Division of Nephrology, Icahn School of Medicine at Mount Sinai, New York, USA

**Keywords:** Alport syndrome, podocyte, therapeutic targets

## Introduction

Alport syndrome (AS) is a heredity disease caused by mutations in the COL4A3/4/5. The disease involves multiple organs, including the kidney, eye, ear, etc. A recent study showed that the predicted pathogenic mutation frequency of COL4A3/4 was about 1/106 and was 1/2320 in COL4A5, suggesting the underestimated prevalence of AS.^[1]^ To improve monitoring and treatment, the Alport Classification Working Group recommended that diseases caused by COL4A3/4/5 mutations be uniformly classified as AS, and the designation of TBMN and COL4A5 “mutation carriers” be removed.^[2]^ High-throughput sequencing further broadens the disease spectrum corresponding to COL4 mutation. It can also be seen in thin basement membrane nephropathy, focal segmental glomerulosclerosis, IgA nephropathy, and diabetic kidney disease.^[3,4,5]^

Hemizygous male and COL4A3/4 recessive inheritance patients have a 100% risk of progression to ESRD. Heterozygous female subjects have a risk to ESRD of approximately 25%. Renin-angiotensin-aldosterone system inhibitor has been the only standard medication for treating AS patients. The poor prognosis of this disease urgently needs more treatment.^[2]^ As a terminally differentiated cell, persistent podocyte damage results in irreversible kidney impairment. Since the podocyte is primarily responsible for synthesizing the COL4A3/4/5 trimer in glomerular basement membrane (GBM), it plays a major role in developing this disease.^[6]^ Until now, no specific drugs targeting podocytes have been developed since the pathogenesis of podocyte injury remains unclear. Here, we summarized the progress on mechanism research of podocytes in AS and the prospect for new therapeutic targets.

## Glomerular basement membrane defection

Podocytes synthesize and secrete COL4A3/4/5 trimers predominantly in the kidney. Impaired trimer formation and GBM defection are two of the fundamental mechanisms contributing to AS progression.^[7]^ To address this issue, editing mutated genes or introducing normal genes using the CRISPR/Cas9 technique or Antisense-oligonucleotides (ASO) has been conducted to prevent or slow disease progression.

Daga *et al*. have successfully corrected Col4A3 and Col4A5 mutations in urine-derived podocytes using dual-plasmid CRISPR/Cas9 gene editing. However, this approach still needs to be verified in future *in vivo* studies.^[8]^ Another study showed an exon-skipping therapy using ASO to reduce disease severity in male X-linked AS.^[9]^ This therapy selectively skipped a targeted exon containing a premature termination codon mutation and corrected the open reading frame. As a result, exon-skipping therapy can re-express the COL4α5 chain on glomerular and tubular basement membranes, significantly reducing proteinuria and prolonging survival in AS mice. In summary, CRISPR/Cas9 gene editing could completely correct all kinds of mutation in podocyte and therefore synthesize normal COL4α3/4/5 trimers, but concerns exist about off-target effect and lack of *in vivo* validation. ASO therapy to skip the exon where the mutation is located, was used in truncated COL4α3/4/5 resulting from nonsense or splicing mutations. Although this is not a complete recovery, it can significantly improve the patients’ prognosis by changing the mutation types.

In some cases, COL4A3/4/5 are truncated due to nonsense variants or premature termination codons (PTCs). Drug-induced PTC readthrough is a promising therapeutic strategy and has been well studied in other genetic diseases like cystic fibrosis and duchenne muscular dystrophy.^[10,11]^ Excitingly, a study showed that 11 nonsense variants from AS patients were highly sensitive to aminoglycoside-mediated PTC readthrough, suggesting a fraction of patients could benefit from such therapy.^[12]^ ELX-02 is a small-molecule eukaryotic ribosomal selective glycoside acting to induce read-through of PTCs. A Phase 2 open label pilot study to evaluate the safety and efficacy of ELX-02 in patients with X-linked or autosomal recessive AS with Col4A3/4/5 nonsense mutation is currently recruiting patients.

Studies have shown that injecting healthy mouse or human embryonic stem cells into COL4 α 3 knockout mice promoted the re-synthesis of the COL4 α 3 chain, reshaped the GBM structure, and eventually improved the renal function of AS mice.^[13]^ Our group demonstrated that intravenous injection of human umbilical cord mesenchymal stem cells into COL4A3 mutant mice attenuated proteinuria and renal pathological damages, and the expression of the COL4A3 chains was also restored to some extent.^[14]^ Stem cell therapy might be a promising treatment option for AS patients in the future.

## Unfold protein reaction and endoplasmic reticulum stress

Unfold protein reaction (UPR) promotes the synthesis of molecular chaperones involving protein folding that prevents cells from stress at an early stage. However, the mutations resulting in persistent UPR could become maladaptive and cytotoxic.^[15]^ Endoplasmic reticulum stress (ERS) has been discussed in many kidney diseases, including diabetic nephropathy, acute kidney injury, and chronic kidney disease.^[15,16]^ COL4A3/4/5 mutations cause abnormal or defective proteins that fail to fold correctly. These misfolded proteins accumulate in the ER, leading to UPR and ERS.^[17,18]^ One of our previous studies also indicated that MG132 (a proteasome inhibitor) intervention improved ERS-related apoptosis in podocytes with truncated COL4A3 mutation.^[19]^ A study in fibroblast cell lines of men with XLAS further showed Sodium 4-phenylbutyrate enhanced COL4α5 transcript levels, reduced ERS, and possibly facilitated COL4A5 extracellular transport.^[20]^ Thus, some molecular chaperone molecules that are able to alleviate ER stress in podocytes might be a promising therapeutic approach for delaying AS progression.

## Cell cycle reentry

The cell cycle is composed of Gap 1 (G1), DNA synthesis (S), Gap 2 (G2), and mitosis (M). The progression of cells through different phases of the cell cycle is mediated by cyclins and cyclin - dependent kinases (CDKs). Manipulating CDKs like CDK4/6 inhibitors has been used in halting tumor growth.^[21]^ However, these approaches have not been thoroughly studied in glomerular disease. Research has revealed that increasing detachment of podocytes was detected in AS patients’ urine. Ding *et al*. reported that the podocyte detachment rate increased 11-fold in the mutant compared to the control condition, and glomeruli lost an average of 26 podocytes per year in AS versus the controls, which equals to 2.3 podocytes per year.^[22]^ After loss of podocytes in glomeruli, remaining podocytes reenter the cell cycle to become hypertrophy, which may help to cover more area on the GMB as compensation.^[23]^ Using proteomic analysis, Frank *et al*. found an increasing fraction of podocytes in G1 or later cell cycle stages in XLAS mice. The podocyte becomes hypertrophic with the transition from G0 to G1.^[24]^ Another study showed a significant decrease in cyclin kinase inhibitors (p27, P21) expression in the kidneys of children with AS.^[25]^ Therefore, cell cycle dysregulation may contribute to the pathogenesis of AS.

## Lipid metabolism

Numerous studies proposed that cholesterol deposition plays a vital role in Alport nephropathy. Wright *et al*. showed that Col4a3 knockout mice exhibited significantly elevated cholesterol accumulation in glomeruli and decreased expression of ABCA1, a gene related to cholesterol efflux and transport.^[26]^ Non-selective cholesterol scavenger hydroxypropyl-β-cyclodextrin (HPβCD) mitigates renal cortex cholesterol accumulation, reduces proteinuria, and alleviates renal tissue inflammation and fibrosis.^[26]^ Sterol-O-acyltransferase-1 (SOAT1) can convert free cholesterol to cholesteryl esters. Inhibition of SOAT1 reduces cholesterol esters in podocytes and promotes free cholesterol efflux through upregulation of ABCA1, thereby attenuating renal damage in AS mice.^[27]^ Another study also found that COL1-mediated discoid domain receptor 1 (DDR1) activation in Col4α3 knockout mice promoted fatty acid uptake by podocytes *via* CD36 and triggered cellular lipo-toxicity. ^[28]^ Thus, drugs that reduce serum cholesterol level such as Ezetimibe, Atorvastatin or small molecules that target ABCA1, SOAT1, and CD36 could be potential approach to treat AS. Sodium-glucose cotransporter-2 inhibitors (SGLT2i) empagliflozin has shown its potential in treating AS mice model *via* switching energy substrates from glucose to fatty acids in podocytes, leading to downregulated lipo-toxicity and improved kidney.^[29]^ Excitingly, a phase 3 clinical trial with Dapagliflozin (NCT05944016) is starting to recruit AS in children and young adults at the early stages of the disease. Altogether, these data indicate that designing drugs targeting lipid metabolism and mitochondria is a meaningful direction for AS treatment.

## Abnormal cell signaling

Furthermore, COL4α3/4/5 defects resulted in sustained expression of the COL4α1/1/2 trimer in GBM, which exists only in the developmental stages of the kidney.^[7]^ Abnormal collagen deposition causes podocyte injury by activating collagen receptors, like DDR1 and integrin α2β. Integrin α2β1 deletion attenuated glomerulosclerosis and interstitial fibrosis in AS mice.^[30]^ Loss of collagen-receptor DDR1 delayed renal fibrosis and reduced inflammation of AS mice.^[31]^ However, selective DDR1 or Integrinα2β1 antagonist has not been developed.

Additionally, endothelin-1 (ET-1) produced by glomerular endothelial cells binds to endothelin A receptors expressed in mesangial cells, causing Laminin α2β1γ1 secretion and deposition in GBM. Ectopic Laminin α2 deposition in GBM activates nuclear factor ϰB (NF-ϰB), stimulates podocyte focal adhesion kinase, and increases the expression of matrix metalloproteinases and proinflammatory factors.^[32,33]^ A Phase 2 clinical trial (AFFINITY) has been initiated to assess the efficacy and safety of Atrasentan, a selective endothelin receptor antagonist, in patients with proteinuric glomerular disease, including 20 patients with AS.

## Oxidative stress and inflammation

Finally, oxidative stress and subsequent activation of inflammation are also important factors contributing to the development of podocyte injuries in Alport syndrome. Nuclear factor erythroid2-related factor2 (Nrf2) has recently been regarded as a major role in regulating cellular oxidative stress in podocytes. Under stress conditions, Keap1 separated from Nrf2, and Nrf2 transferred to the nucleus, promoting the expression of genes carrying antioxidant response elements and initiating antioxidation.^[34,35]^ Nrf2 also suppresses transcription of inflammation-related genes by inhibiting NK-ϰB activation.^[35]^ A Phase 3 clinical trial (CARDINAL) has been conducted to investigate the safety and efficacy of bardoxolone in treating patients with AS. However, the trial’s outcomes were unsatisfactory.^[36]^ NOX4 is the major renal reactive oxygen species (ROS) source and is abundantly expressed in podocytes.^[37]^ Our earliest research found that NOX4 and ROS were significantly upregulated in patients with ADAS, and increased MMP-2 and podocyte apoptosis could be rescued by NOX4 inhibition *in vivo* and in vitro.^[38]^ A recent study demonstrated that Mineralocorticoid receptor antagonist finerenone protected Col4a3-/- mice by suppressing the residual interstitial inflammation and fibrosis,^[39]^ further studies are required to pinpoint finerenone’s precise mechanism on podocytes. We speculate that drugs targeting NF-kB activation and reducing ROS production in podocytes will effectively improve patients’ prognosis.

## Conclusion and perspective

**Figure 1:**
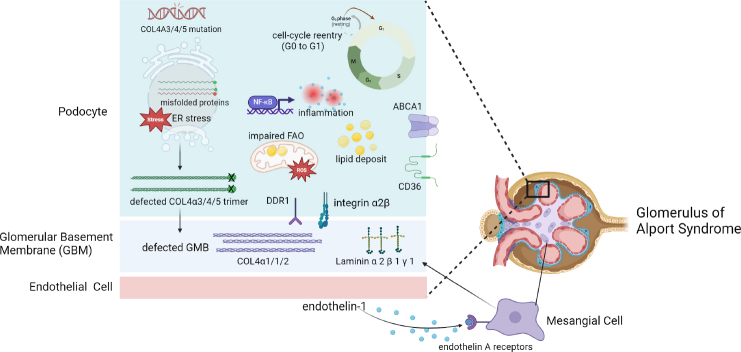
Pathogenic mechanisms of intrinsic cells and basement membrane in glomerulus of Alport syndrome. Created with BioRender.

To summarize, the podocyte injury in AS is primarily caused by ERS, cell cycle dysregulation, lipid toxicity, inflammation, oxidation stress, and abnormal downstream cell signaling activation (Figure 1). Enabling podocytes to express the correct COL4A3/4/5 through gene editing or transferring the corrected COL4A3/4/5 gene into podocytes might be the most effective way to cure the disease. Delaying the onset of the disease and reducing the disease progression are also essential for management of this disease. Preventing the generation of truncated proteins by exon skipping or PTCs readthrough therapy, Reducing ERS by molecular chaperone, decreasing cell detachment by modification of cell cycle, promoting lipid metabolism or cholesterol efflux in podocyte, and inhibiting inflammatory response and oxidative stress are all potential therapeutic approaches to ameliorate podocyte injury in AS.
